# Similar responsiveness between C57BL/6N and C57BL/6J mouse substrains to superovulation

**DOI:** 10.17912/micropub.biology.000375

**Published:** 2021-02-25

**Authors:** Miyuki Shindo, Hideki Tsumura, Kenji Miyado, Woojin Kang, Natsuko Kawano, Tomoko Yoshida, Maki Fukami, Mami Miyado

**Affiliations:** 1 Division of Laboratory Animal Resources, National Research Institute for Child Health and Development, 2-10-1 Okura, Setagaya, Tokyo 157-8535, Japan; 2 Department of Reproductive Biology, National Research Institute for Child Health and Development, 2-10-1 Okura, Setagaya, Tokyo 157-8535, Japan; 3 Laboratory of Regulatory Biology, Department of Life Sciences, School of Agriculture, Meiji University, 1-1-1 Higashimita, Kawasaki, Kanagawa 214-8571, Japan; 4 Department of Molecular Endocrinology, National Research Institute for Child Health and Development, 2-10-1 Okura, Setagaya, Tokyo 157-8535, Japan

## Abstract

Superovulation is a method for the drug-induced release of multiple eggs and useful for *in vitro* fertilization. Thus, its high efficiency largely reduces the number of mice used per experiment. We compared the responsivity to superovulation between C57BL/6N (B6N) and C57BL/6J (B6J) substrains. The average number of ovulated eggs was strikingly higher in both substrains treated with anti-inhibin serum (AIS) plus equine chorionic gonadotropin (eCG) than those treated with eCG alone. Our data indicate that hypothalamus–pituitary–ovarian axis similarly responds to eCG treatment in B6N and B6J mice, and that this responsiveness is enhanced by the presence of AIS.

**Figure 1:**
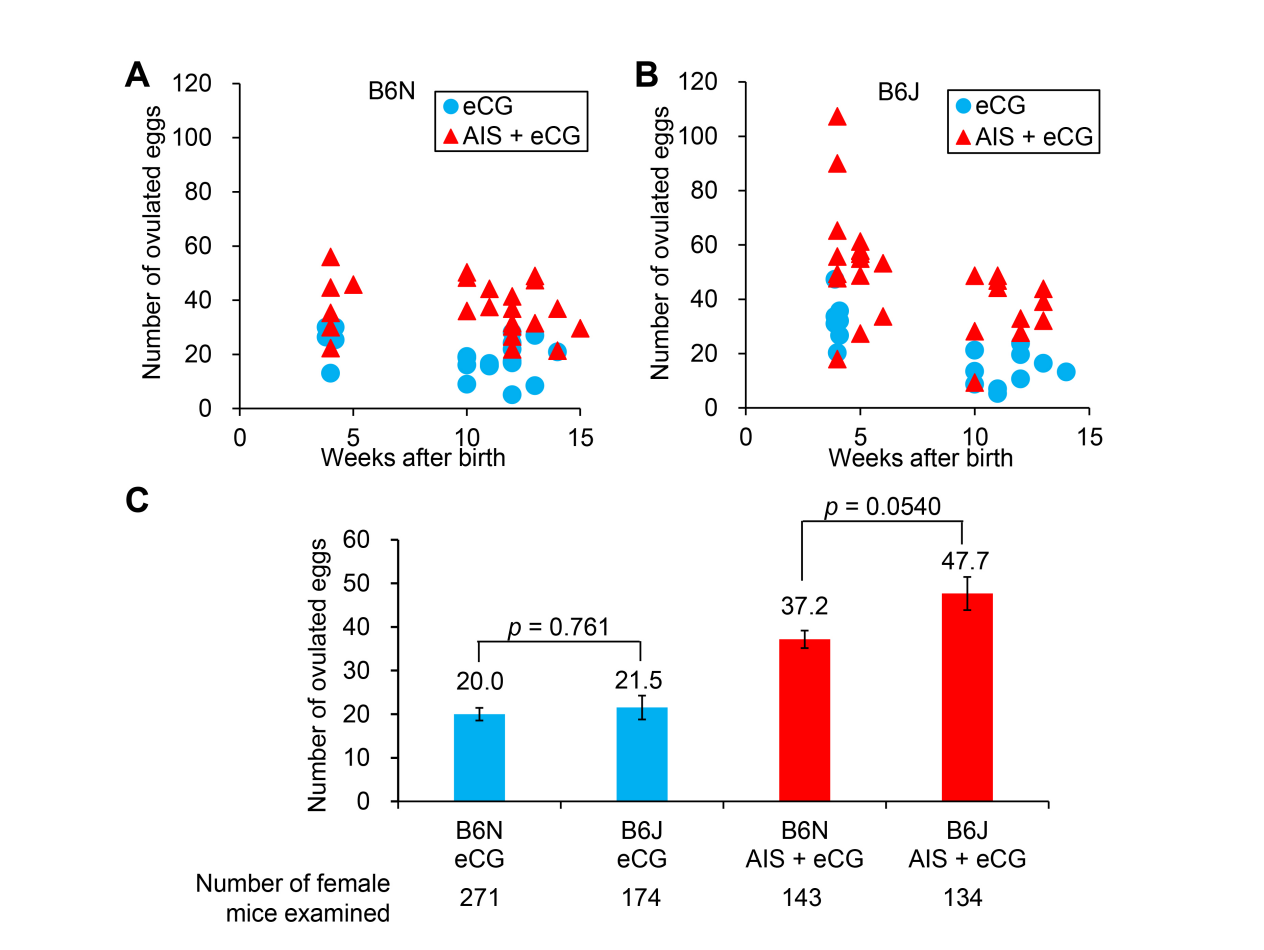
**Comparison of the responsivity of C57BL/6N (B6N) and C57BL/6J (B6J) mice to superovulation.** Average number of ovulated eggs from B6N (A) and B6J (B) mice treated with equine chorionic gonadotropin (eCG) alone or anti-inhibin serum (AIS) plus eCG. Each symbol (circle, eCG alone; triangle, AIS + eCG) includes a group of three or more female mice. (C) The number of ovulated eggs between B6N and B6J mice treated with eCG or AIS + eCG. Values are expressed as mean ± standard error of the mean.

## Description

Superovulation refers to the drug-induced release of multiple eggs that is required for *in vitro* fertilization (IVF), and its high efficiency largely reduces the number of mice used per experiment. Both follicle stimulating hormone (FSH) and pregnant mare’s serum gonadotropin also known as equine chorionic gonadotropin (eCG) stimulate ovarian follicular development and inhibin secretion (Fowler and Edwards 1957). Inhibin has been isolated from ovarian follicular fluid as a substance that selectively inhibits FSH secretion from the pituitary gland (Luisi **et al.** 2005). Inhibin suppresses ovulation, and its function is neutralized by anti-inhibin serum (AIS) (Takeo and Nakagata 2015). Treatment with AIS plus eCG induced the ovulation of higher number of eggs than eCG alone in C57BL/6J (B6J) mice aged between 3 and 30 weeks, except for 6 weeks of age (Takeo **et al.** 2019).

One of the commonly used inbred strains of mice, C57BL/6, has been divided into multiple substrains. C57BL/6N (B6N) and B6J mice have been separated for more than 50 years. Their genetic differences are negligible, but there are notable differences in metabolic responses, such as obesity (Ge **et al.** 2019), glucose tolerance and insulin secretion (Fergusson **et al.** 2014; Wong **et al.** 2010), and responses to hypoxic-ischemic brain injury (Wolf **et al.** 2016). These findings should deter future use of mice with mixed between B6 substrains in basic research, especially when studying endocrine systems. Since the secretion of several endocrine hormones is regulated via the hypothalamus–pituitary–ovarian axis (Baird 1987), these phenotypic differences between B6N and B6J mice may occur by an alteration in function of the hypothalamus–pituitary–ovarian axis.

In this study, we compared the responsivity to superovulation between B6N and B6J substrains (Figure 1). Female mice were treated with either eCG alone or AIS plus eCG (Takeo and Nakagata 2015), followed by human chorionic gonadotropin (hCG) treatment. The average number of ovulated eggs was strikingly higher in both mouse substrains treated with AIS plus eCG than those treated with eCG alone (Figure 1A, B). The average number of ovulated eggs at 4 weeks of age becomes maximum (over 100 eggs) by treatment with AIS plus eCG in B6J mice (Figure 1B) as described previously (Takeo **et al.** 2019), while the strikingly increased number of ovulated eggs at 4 weeks of age was unseen in B6N mice (Figure 1A). When mice were treated with eCG alone, the average number of ovulated eggs was comparable between B6N and B6J mice (20.0 ± 1.5 and 21.5 ± 2.7, respectively; *p* = 0.761) (Figure 1C). Similarly, when they were treated with AIS plus eCG, the average number of ovulated eggs was comparable between B6N and B6J mice (37.2 ± 2.0 and 47.7 ± 3.8; *p* = 0.0540) (Figure 1C). Data at 4 weeks of age showed no significant difference between B6N and B6J mice with AIS (37.7 ± 5.2 and 61.9 ± 10.3; *p* = 0.118) and without AIS (26.2 ± 2.2 and 32.4 ± 2.9; *p* = 0.073). As reported previously (Takeo **et al.** 2019; Takeo and Nakagata 2015), when the eggs collected from B6N and B6J female mice were subjected to IVF, the fertilization rate of eggs was unaffected by the presence or absence of AIS. These results suggest that responsivity to superovulation treatment is similar between B6N and B6J mice.

In the present study, we showed that B6N and B6J mice similarly respond to superovulation treatment. However, a single increased peak of ovulated eggs was observed in B6J mice but not in B6N mice at 4 weeks of age when AIS was present. This finding implies that the responsivity of hypothalamus–pituitary–ovarian axis in B6N mice differs from that in B6J mice at least at 4 weeks of age. In other words, B6J mice probably carry the hypothalamus–pituitary–ovarian axis with high responsivity at 4 weeks of age. As the results obtained from this study are consistent with those of a previous study using B6J mice (Takeo **et al.** 2019), we were able to reproduce the experimental results at other facilities. On the other hand, one group of B6J mice showed the average number of ovulated eggs (nearly 20) at 4 weeks of age despite the presence of AIS, indicating that responsiveness against treatment with AIS plus eCG in B6J mice depends on individual differences. However, regardless of the presence or absence of AIS, ratios of females without response were almost the same between these two substrains at 4 weeks of age.

We conclude that hypothalamus–pituitary–ovarian axis similarly responds to eCG treatment in B6N and B6J mice, and that this responsiveness is enhanced by the presence of AIS. Our data, together with previous studies (Takeo **et al.** 2019; Takeo and Nakagata 2015), indicate that the increased number of ovulated eggs largely contributes to a reduction in the number of mice required for animal studies.

## Methods

**Animals**

The B6N and B6J mice were purchased from Japan SLC Inc. (Shizuoka, Japan). All mice were housed under specific pathogen-free conditions with access to food and water *ad libitum*, and controlled conditions of room temperature at 23 ± 1ºC and 12 h light-dark cycles (daily light period, 0800 to 2000 h). All animal experiments were approved by the Institutional Animal Care and Use Committee of the National Research Institute for Child Health and Development (Experimental number, A2005-007).

**Superovulation**

Female B6N and B6J mice (4–15 weeks of age) received intraperitoneal injections of 7.5 IU of eCG (ASKA Animal Health, Tokyo, Japan) or AIS plus eCG (0.1 mL CARD HyperOva; Kyudo, Saga, Japan), followed by 7.5 IU of hCG (ASKA Animal Health) after 48 hours. Eggs at metaphase II stage were collected from oviducts of female mice 14–16 hours after administration of hCG, and their numbers were counted under a stereoscope (SMZ645; Nikon, Tokyo, Japan) as previously described (Shindo **et al.** 2019).

**Statistical analysis**

Low or no-responsive females were included in this analysis. Values are expressed as mean ± standard error of the mean. Significant differences (*p*-values) between B6N and B6J mice were calculated by performing a Student’s *t*-test or a Mann-Whitney’s *U*-test. A *p*-value of less than 0.05 was considered significant.
